# A Case of Traumatic Tetanus—Recovery

**Published:** 1880-01

**Authors:** Allison Maxwell


### A CASE OF TRAUMATIC TETANUS—RECOVERY.

By ALLISON MAXWELL, M.D.

Albert Mathers, aged nine, was brought to me August 6, 1870. He was led by his father, and on attempting to sit down had some opisthotonus, so that he had to make a second effort before he became seated. Thirteen days before Albert had the last phalanx of the index finger of the right hand almost mashed off by a printing press. The finger was dressed by my father, Dr. J. D. Maxwell, of Bloomington, Ind., and was healing rapidly when the boy returned to his home in this city. On the ninth day after the injury he received a blow on the finger, which caused profuse bleeding. That evening at supper he complained of some stiffness of the jaws, and during the night was quite restless. The next morning the muscles of the jaw were more rigid, deglutition difficult, and there was some sore throat. A physician in the neighborhood prescribed a liniment to be rubbed on his jaws, but without benefit. Patient slept but little the following night; complained of stiffness in the muscles of the back of neck, and the night before I saw him he had slept still less. His appetite has continued good, though he is unable to eat solid food. He walks with difficulty. On asking to see his tongue the patient took a stick out of his pocket and pried his teeth apart. I prescribed eight grains of chloral hydrate every four hours, and when I saw him in the afternoon put some powdered chloral on the injured finger, as recommended by Dr. J. K. Bigelow in the *American Practitioner*. The smart of the application was so severe, however, that it could not be borne; nor was he subsequently willing to endure the pain caused by even a weak solution of chloral to the wound. On attempting to move him he would have an attack of trismus and opisthotonus. Pulse 80, temperature 101°. Having slept none during the day, the chloral was ordered every two hours.

*Fourteenth day.*—Very restless all night; slept none; this morning convulsions violent and almost continuous. The muscles of the abdomen seemed to remain in tonic spasm. I now gave one fourth grain morphia hypodermically, and directed the chloral to be given every hour. Patient became more quiet after the morphia, and slept at intervals from ten to thirty minutes at a time. Pulse 120, temperature 103°. He bit his tongue several times to-day in spite of the usual precautions. Increased the dose of chloral in the afternoon to ten grains.

*Fifteenth day.*—Slept during night four or five hours; is better this morning. During the afternoon could flex his legs, but could not separate the jaws more than a line.

*Sixteenth day.*—Marked improvement in all respects, but still requires the ten grains of chloral hourly to prevent convulsions.

*Seventeenth day.*—Rested well last night under the chloral. Pulse 100, temperature 100°.

*Eighteenth day.*—The symptoms now gradually abated, and the dose of chloral was daily decreased, although patient often complained of cramp of the diaphragm and abdominal muscles.

On the twenty-third day after the tetanus began there appeared over the whole body an eruption resembling that of measles, but more elevated and redder, and accompanied by much itching. Pulse was now 90 and temperature normal. The eruption seemed to be fairly attributable to the chloral which had been and was still being given; for when this drug was diminished the eruption faded, and when the dose was increased the eruption and the itching returned. The patient was able to take liquid nourishment throughout his attack.

The prognosis in this case was very doubtful; first, on account of the severity of the spasms: second, on account of the symptoms beginning before the tenth day; and third, on account of the boy's age.

While an interne in the Cincinnati Hospital I saw two severe cases of traumatic tetanus recover—one under chloral alone, the other under chloral and hypodermic morphia and atropia. Chloral hydrate is clearly taking high rank as a remedy for tetanus. Many recent authors prefer it to all other remedies. Bauer, in Ziemssen's Cyclopedia, says chloral is superior to every other means for holding in check the spasms. Hamilton on Nervous Diseases considers that chloral has been the most efficacious remedy in tetanus. Flint, in his Clinical Medicine, states that chloroform and chloral are the most potent remedies in this disease, preferring the former as being more manageable. The London *Lancet* during the last six months has contained reports of six or eight cases of tetanus treated with chloral, and in which the percentage of recoveries seems unusually large.—*Am. Practitioner.*

